# Association between air pollutants, thyroid disorders, and thyroid hormone levels: a scoping review of epidemiological evidence

**DOI:** 10.3389/fendo.2024.1398272

**Published:** 2024-10-08

**Authors:** Kaijie Yang, Guofeng Zhang, Yongze Li

**Affiliations:** Department of Endocrinology and Metabolism, Institute of Endocrinology, National Health Commission (NHC) Key Laboratory of Diagnosis and Treatment of Thyroid Disease, The First Hospital of China Medical University, Shenyang, China

**Keywords:** air pollutants, scoping review, thyroid disease, epidemiology, atmospheric particulate matter

## Abstract

**Background:**

Over the past two decades, the incidence of thyroid disorders has been steadily increasing. There is evidence to suggest that air pollution may be one of the etiological factors of thyroid diseases. This comprehensive review aimed to examine the evidence related to air pollutants and thyroid disorders and thyroid hormones levels from an epidemiological perspective.

**Methods:**

The scoping review adopted a systematic approach to search for, identify, and include peer-reviewed articles published in English. We performed a comprehensive search of three databases-PubMed, Embase, and Web of Science to identify relevant literature on the relationship between air pollution [particulate matter, nitrogen oxide, carbon monoxide (CO), ozone (O_3_), sulfur dioxide (SO_2_)] exposure and thyroid disorders, including hypothyroidism, congenital hypothyroidism (CH), thyroid nodules, thyroid cancer, autoimmune thyroid diseases, as well as thyroid hormone levels, such as thyroid-stimulating hormone (TSH), free triiodothyronine (FT_3_), and free thyroxine (FT_4_). Articles published until August 1, 2023, were included.

**Results:**

A total of 3,373 studies were retrieved, and among them, 25 studies covering eight different air pollutants were relevant. The most frequently studied air pollutants in this review included fine particulate matter (with fine particulate matter (PM_2.5_), n=21; inhalable particles (PM_10_), n=10; PM_10-2.5_, n=1) and nitrogen oxides (with NO_2_, n=13; NOx, n=3). The thyroid disorders and thyroid hormone levels most commonly associated with evidence of air pollution exposure were hypothyroidism (n=7) and TSH (n=12).

**Conclusions:**

Despite variations in study designs and exposure assessments, the findings consistently highlight the substantial health risks that air pollution, particularly PM_2.5_, poses to thyroid health, especially among vulnerable populations. Given that our study was limited to epidemiological investigations and the increasing prevalence of toxic substances in the environment, there is an urgent need for further research to elucidate the mechanisms by which these pollutants disrupt thyroid function and contribute to the development of thyroid diseases.

## Introduction

Air pollution is a globally recognized environmental health hazard (1). With rapid global socioeconomic development, there is growing concern about the adverse health effects of air pollutants (2). Environmental air pollution and indoor air pollution are considered major risk factors leading to premature mortality and an increased incidence of diseases. The burden of diseases and deaths caused by these factors has become a global public health challenge, imposing significant direct and indirect costs on society (3). In 2021, particulate matter air pollution was the leading contributor to the global disease burden in 2021 (4). Air pollutants can be categorized into two main types based on their state of existence: gaseous pollutants and particulate pollutants. Gaseous pollutants include nitrogen dioxide (NO_2_), carbon monoxide (CO), ozone (O_3_), sulfur dioxide (SO_2_), and others. Atmospheric particulate matter (PM) comprises total suspended particles, inhalable particles (PM_10_), fine particulate matter (PM_2.5_), and ultrafine particles. Among these, PM_2.5_ refers to particles in the atmosphere with a diameter equal to or less than 2.5μm. PM_2.5_ represents the majority of PM in the atmosphere (5, 6).

Thyroid disorders have been recognized as some of the most prevalent diseases worldwide (7). Common biomarkers for assessing thyroid homeostasis include free thyroxine (FT_4_), free triiodothyronine (FT_3_), and thyroid-stimulating hormone (TSH) (8). Thyroid hormones (THs) play a pivotal role in maintaining metabolic balance, cardiovascular health, and neurological development, exhibiting pleiotropic effects on multiple organ systems (9). Abnormal TH levels, whether elevated or decreased, are closely associated with various thyroid disorders (10). While iodine nutrition status is significantly linked to thyroid disease, research indicates that the prevalence of thyroid disorders continues to rise in iodine-sufficient populations, such as in China (11). Furthermore, the global incidence of thyroid disease is also on the rise (12–14). Animal studies and extensive epidemiological research suggest that air pollutants can disrupt thyroid hormone levels, impair metabolic homeostasis, and ultimately contribute to thyroid dysfunction (15, 16). Epidemiological studies often utilize large population samples, enabling researchers to observe the effects of air pollutants in real-world settings. Additionally, long-term epidemiological investigations help establish temporal associations between exposure and disease onset. However, establishing a causal relationship between exposure and disease requires further experimental evidence.

This review aims to retrieve and synthesize published epidemiological studies on the relationship between air pollutant exposure and thyroid diseases, as well as thyroid hormones, across various populations, including children, adults, and pregnant women. The findings are interpreted from an epidemiological perspective, offering theoretical insights and directions for future systematic research, and providing new perspectives on the prevention of thyroid diseases.

## Methods

We conducted a scoping review to facilitate the mapping of the literature on emerging topics and provide avenues for future research. Our aim was to gain a comprehensive understanding of the literature regarding the relationship between exposure to air pollutants and thyroid disorders and thyroid hormone levels. Our findings were reported using the Preferred Reporting Items for Systematic Reviews and Meta-Analyses Extension for Scoping Reviews (PRISMA-ScR) (17). The scoping review protocol was registered with the Open Science Framework (https://doi.org/10.17605/OSF.IO/V8ERP).

### Data sources and search strategy

The preliminary search was conducted on PubMed to identify relevant MeSH terms and keywords. Subsequently, a comprehensive systematic search strategy was developed for the PubMed, Embase, and Web of Science databases using the identified keywords and indexing terms (**Tables 1**, **2**). The final literature retrieval was carried out on August 1, 2023.

**Table 1:** Index terms and keywords used for the literature search.

Theme	Search
Thyroid Diseases	Disease, Thyroid OR Diseases, Thyroid OR Thyroid Disease
OR	
Thyroid Hormones	Hormones, Thyroid OR Thyroid Hormone OR Hormone, Thyroid
AND	
Air Pollution	Air Pollutions OR Pollution, Air OR Air Quality
OR	
Nitrogen Dioxide	Dioxide, Nitrogen OR Nitrogen Peroxide OR Peroxide, Nitrogen
OR	
Ozone	Tropospheric Ozone OR Ozone, Tropospheric OR Low Level Ozone OR Level Ozone, Low OR Ozone, Low Level OR Ground Level Ozone OR Level Ozone, Ground OR Ozone, Ground Level
OR	
Sulfur Dioxide	Sulfurous Anhydride
OR	
Nitrogen Oxides	Oxides, Nitrogen OR Nitrogen Oxide OR Oxide, Nitrogen
OR	
Carbon Monoxide	Monoxide, Carbon
OR	
Particulate Matter	Ultrafine Fibers OR Ultrafine Fiber OR Fiber, Ultrafine OR Airborne Particulate Matter OR Particulate Matter, Airborne OR Air Pollutants, Particulate OR Particulate Air Pollutants OR Ambient Particulate Matter OR Particulate Matter, Ambient OR Ultrafine Particulate Matter OR Particulate Matter, Ultrafine OR Particles, Ultrafine OR Ultrafine Particle OR Particle, Ultrafine

**Table 2:** Example of full search strategy in PUBMED.

	Search terms
1	“Thyroid Diseases”[Mesh] OR Disease, Thyroid [Title/Abstract] OR Diseases, Thyroid [Title/Abstract] OR Thyroid Disease [Title/Abstract]
2	“Thyroid Hormones”[MeSH Terms] OR “hormones thyroid”[Title/Abstract] OR “thyroid hormone”[Title/Abstract] OR “hormone thyroid”[Title/Abstract]
3	“Air Pollution”[Mesh] OR Air Pollutions [Title/Abstract] OR Pollution, Air [Title/Abstract] OR Air Quality [Title/Abstract]
4	“Carbon Monoxide”[Mesh] OR Monoxide, Carbon [Title/Abstract]
5	“Nitrogen Oxides”[Mesh] OR Oxides, Nitrogen [Title/Abstract] OR Nitrogen Oxide [Title/Abstract] OR Oxide, Nitrogen [Title/Abstract]
6	“Sulfur Dioxide”[Mesh] OR Sulfurous Anhydride [Title/Abstract]
7	“Ozone”[Mesh] OR Tropospheric Ozone [Title/Abstract] OR Ozone, Tropospheric [Title/Abstract] OR Low Level Ozone [Title/Abstract] OR Level Ozone, Low [Title/Abstract] OR Ozone, Low Level [Title/Abstract] OR Ground Level Ozone [Title/Abstract] OR Level Ozone, Ground [Title/Abstract] OR Ozone, Ground Level [Title/Abstract]
8	“Nitrogen Dioxide”[Mesh] OR Dioxide, Nitrogen [Title/Abstract] OR Nitrogen Peroxide [Title/Abstract] OR Peroxide, Nitrogen [Title/Abstract]
9	Particulate Matter [MeSH Terms] OR Ultrafine Fibers [Title/Abstract] OR Ultrafine Fiber [Title/Abstract] OR Fiber, Ultrafine [Title/Abstract] OR Airborne Particulate Matter [Title/Abstract] OR Particulate Matter, Airborne [Title/Abstract] OR Air Pollutants, Particulate [Title/Abstract] OR Particulate Air Pollutants [Title/Abstract] OR Ambient Particulate Matter [Title/Abstract] OR Particulate Matter, Ambient [Title/Abstract] OR Ultrafine Particulate Matter [Title/Abstract] OR Particulate Matter, Ultrafine [Title/Abstract] OR Ultrafine Particles [Title/Abstract] OR Particles, Ultrafine [Title/Abstract] OR Ultrafine Particle [Title/Abstract] OR Particle, Ultrafine [Title/Abstract]
10	1 OR 2
11	3 OR 4 OR 5 OR 6 OR 7 OR 8 OR 9
12	10 AND 11

### Inclusion and exclusion criteria

The inclusion criteria for the literature were as follows: (1) the exposure factor studied was air pollutants as the primary focus; (2) the literature examined outcomes related to thyroid diseases and thyroid hormone levels; and (3) the literature included results from epidemiological studies.

Exclusion criteria for the literature were as follows: (1) literature that did not meet the inclusion criteria; (2) literature investigating exposure factors such as organic pollutants or chemical substances, among others; (3) duplicate literature, reviews, meta-analyses, letters, replies, comments, or meeting abstracts; and (4) literature for which full text was unavailable, and data extraction was not possible.

### Data extraction

Two researchers conducted a full-text screening, independently reviewed the literature included in the final selection, and extracted the data into tables for the purpose of data visualization, data synthesis, and result reporting. Discrepancies arising during this process were resolved through discussions involving all the authors. For studies meeting the inclusion criteria, we extracted information on the author, year of study, study type, country, study period, study population, sample size, pollutants, pollutants exposure evaluation, pollutants exposure time, thyroid related outcomes. Due to heterogeneity across studies and insufficient support for aggregation of the results, a meta-analysis was not conducted.

## Results

**Figure 1:**
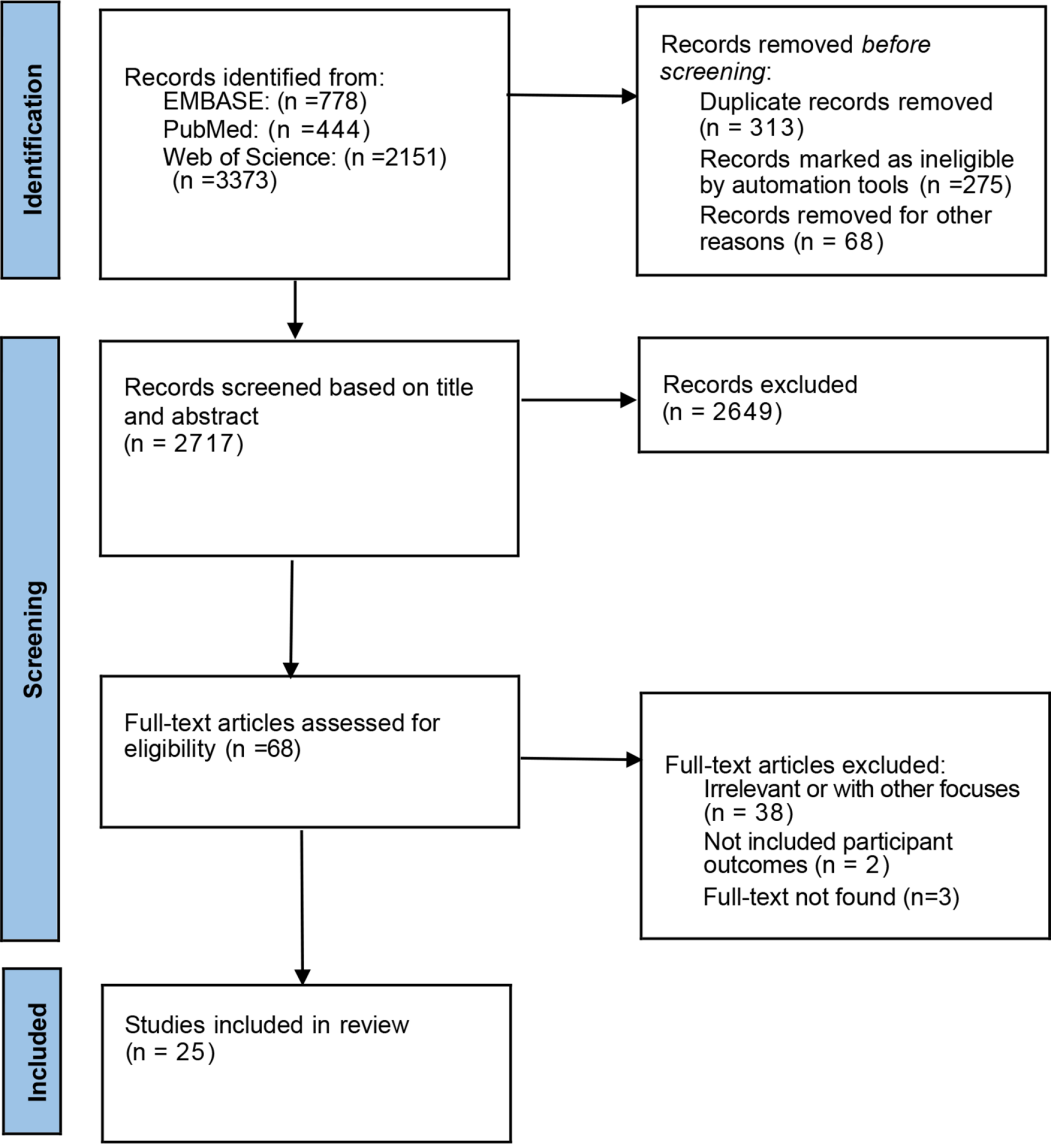
PRISMA flow diagram of study selection.

A total of 3,373 articles related to air pollutants and thyroid diseases were identified in the search. After removing duplicates (313 articles) and performing the initial screening of titles and abstracts, 68 articles remained. Upon further full-text examination and the exclusion of articles not meeting the criteria, 25 articles were considered relevant. **Figure 1** illustrates the Preferred Reporting Items for Systematic Reviews and Meta-Analyses (PRISMA) flowchart for the selection of included studies (**Figure 1**).

### Study characteristics

The included literature consists of population-based epidemiological studies. Most studies employed a cohort study design (12 studies), followed by cross-sectional studies (8 studies), case−control studies (4 studies), and one Mendelian randomization study (**Table 3**). All the included articles were published within the past ten years, with nearly 70% published after 2020. Among the 25 studies, more than half were conducted in Asia, including 12 from China. This study examines eight different air pollutants (PM_2.5_, PM_10_, PM_10-2.5_, O_3_, NO_2_, NOx, SO_2_, CO) and twelve thyroid-related outcomes, including TSH (n=12), FT_4_ (n=10), FT_3_ (n=7), hypothyroidism [n=7, of which 3 are congenital hypothyroidism (CH)], and thyroid cancer (n=3), among others. The 25 studies primarily focus on pregnant women and newborns, with 10 studies involving pregnant women, 7 studies involving newborns, and 6 studies involving the general adult population.

**Table 3:** Association between air pollutants and thyroid disorders.

Author	Country (City)	Study period	Study population	Sample size	Pollutants	Exposure evaluation	Exposure time	Outcomes
Cohort study								
Janssen et al. (18), 2017	Belgium	2010.02-2014.06	Pregnant woman; newborn	431;498	PM_2.5_	Spatial-temporal interpolation	Third trimester	TSH; FT_4_/FT_3_; FT_4_; FT_3_
Howe et al. (52), 2018	America	1994-1997/2000-2003	Newborn	2050	PM_2.5_; PM_10_; _O3_; NO_2_; NOx	Spatial-temporal interpolation	Whole pregnancy	TT_4_
Wang et al. (19), 2019	China	2014-2015	Pregnant woman; newborn	431 pairs	PM_2.5_ and its six main constituents	Satellite data simulations	First trimester	FT_4_; TSH
Ghassabian et al. (22), 2019	Greece; Netherland; Spain; America	2007.02-2008.02; 2003.01-2004.03; 2003.11-2008.01; 1999.04-2002.01	Pregnant woman	9931	PM_2.5_; PM_10_; NO_2_; NOx	Spatial-temporal interpolation; Satellite data simulations	First trimester	Hypothyroxinemia; High TSH
Zhao et al. (23), 2019	China	2014.04-2015.11	Pregnant woman	8077	NO_2_, PM_2.5_	Spatial-temporal interpolation	First trimester; Second trimester	Hypothyroxinemia; FT_4_; TSH
Li et al. (53), 2021	China	2013.10-2015.07	Pregnant woman	551	PM_2.5_	Spatial-temporal interpolation	First trimester; three months of preconception	TSH; FT_4_/FT_3_; FT_4_; FT_3_
Irizar et al. (54), 2021	Spain	2006-2008	Newborn	463	PM_2.5_, NO_2_	Spatial-temporal interpolation	Whole pregnancy	TT_4_
Harari-Kremer et al. (24), 2021	Israel	2008.01-2015.12	Newborn	696,461	NOx; NO_2_; PM_2.5_; PM_10-2.5_	Spatial-temporal interpolation; Satellite data simulations	Whole pregnancy	CH; TT_4_
Zhou et al. (55), 2022	China	2016.04-2018.12	Pregnant woman	1060	PM_2.5_ and its metal constituents	Satellite data simulations	First trimester	TSH; FT_4_/FT_3_; FT_4_; FT_3_
Zhang et al. (20), 2022	China	2013.01-2014.10	Pregnant woman	921	PM_2.5_, PM_10_	Spatial-temporal interpolation	First trimester	TSH; FT_4_/FT_3_; FT_4_; FT_3_
He et al. (21), 2022	China	2010.01-2019.12	Adult	191,357	O_3_	Monitoring station	Annual average	TNs; T_3_; T_4_
Lzic et al. (25), 2022	Bosnia and Herzegovina	2015.01.01-2020.12.31	AITD patient	174	NO_2_, SO_2_, _O3_, CO, PM_2.5_	Monitoring station	Annual average	AITD
Cross-sectional study								
Shang et al. (30), 2019	China	2014.10.01-2015.10.01	Pregnant woman; newborn	15,100,000	PM_2.5_; PM_10_	Monitoring station	Whole pregnancy	CH
Kim et al. (26), 2020	Korean	2009-2015	Adult	4704	NO_2_; CO; PM_10_; SO_2_;	Monitoring station	Annual average	TSH; FT_4_
Ilias et al. (28), 2020	Athens	2019	Pregnant woman	293	PM_2.5_	Monitoring station	Average preceding nine month	TSH
Zeng et al. (2), 2021	China	2013.12-2018.12	Adult	327,913	PM_2.5_; PM_10_; SO_2_; CO; NO_2_; _O3_	Monitoring station	Annual average	TSH; FT_4_/FT_3_; FT_4_; FT_3_
Zhang et al. (32), 2021	China	2015-2017	Adult	4,920,536	PM_2.5_, PM_10_, NO_2_, SO_2_, CO, _O3_	Monitoring station	Annual average	TNs
Qi et al. (31), 2021	China	2014.01.01-2015.12.30	Newborn	NA	O3, NO_2_, SO_2_, CO	Monitoring station	Annual average	CH
Valdés et al. (29), 2022	Spain	2008-2010	Adult	3859	PM_2.5_, NO_2_	Spatial-temporal interpolation	Annual average	TSH; FT_4_/FT_3_; FT_4_; FT_3_
Qiu et al. (29), 2022	China	2018.01-2018.12	Pregnant woman	2528	PM_2.5_; PM_10_; SO_2_; CO; NO_2_	Monitoring station	First trimester	TSH; FT_4_/FT_3_; FT_4_; FT_3_
Case-control study								
Crepeau et al. (34), 2023	America	2013.01-2016.12	Patients; matched controls without thyroid disease	1,990; 6,919	PM_2.5_	Monitoring station; Spatial-temporal interpolation; Satellite data simulations	12-, 24-, and 36-month average concentration	PTC
Park et al. (33), 2021	Korean	2002-2015	Patients; matched controls	4,632; 18,528	SO_2_; NO_2_; _O3_; CO; PM_10_	Monitoring station	Annual average	THCA
Sun et al. (35), 2023	China	2012-2020	Hypothyroid patients; matched controls	795; 2,385	PM_2.5_, PM_10_	Spatial-temporal interpolation	30, 60, and 90 days preceding the LMPM	Hypothyroidism
Karzai et al. (36), 2022	America	2013-2016	PTC patients; matched healthy controls	1,990; 3,980	PM_2.5_	Monitoring station; Satellite data simulations	12-, 24-, and 36-month average concentration	PTC
Mendelian randomization study								
Zhang et al. (37), 2022	European	NA	Hypothyroidism patients; controls	22,687; 440,246	PM_2.5_	Spatial-temporal interpolation; Satellite data simulations	Annual average	Hypothyroidism

AITD, autoimmune thyroid diseases; CH, congenital hypothyroidism; CI, confidence interval; CO, carbon monoxide; FT_3_, free triiodothyronine; FT_4_,free thyroxine; LMPM, last menstrual period month; NA; not available NO_2_, nitrogen dioxide; NO_X_, nitrogen oxides; O_3_, ozone; OR, odds ratio; PM_2.5_, fine particulate matter; PM_10_, inhalable particles; PM_10–2.5_, particulate matter with aerodynamic diameter of 2.5–10μm; PTC, papillary thyroid cancer; SO_2_, sulfur dioxide; THCA, thyroid carcinoma; TN_S_, thyroid nodules; TSH, thyroid-stimulating hormone; TT_4_, total thyroxine.

### Cohort studies

Out of the 25 studies included, 12 were cohort studies. Of these, 11 addressed thyroid hormone (THs) levels. Research found that, during late pregnancy, exposure to PM_2.5_ was negatively correlated with both umbilical cord blood TSH levels and the FT_4_/FT_3_ ratio (18). Higher PM_2.5_ exposure during pregnancy was associated with decreased maternal FT_4_ levels (19) and a reduced FT_4_/FT_3_ ratio (20). However, some studies have found no statistically significant associations between maternal PM_2.5_ exposure and neonatal TSH concentrations (19). Regarding thyroid disease, one large-scale cohort study spanning a decade among the Chinese population evaluated the relationship between O_3_ and thyroid diseases (TNs). The study indicates that long-term exposure to high levels of O_3_ in Hunan Province may be associated with an increased detection rate of TNs in general adults, potentially mediated by TSH (21). The other three studies focused on hypothyroidism, with one addressing full-term newborns with CH. Ghassabian et al. conducted a cohort study using data from five birth cohorts, comprising a total of 9,931 pregnant women. The study found that exposure to PM_2.5_ in early pregnancy was linked to mild thyroid dysfunction persisting throughout pregnancy. However, exposures to NOx and NO_2_ were not associated with hypothyroxinemia or high TSH during pregnancy (22). Another cohort study conducted in Shanghai, China, reached similar conclusions, reporting that early pregnancy (0-12 weeks) and mid-pregnancy (13-26 weeks) exposure to PM_2.5_ was associated with an increased risk of hypothyroidism in pregnant women. However, there was no significant association between hypothyroidism and NO_2_ exposure (23). An Israeli cohort study demonstrated a positive correlation between late-pregnancy exposure to nitrogen oxide (NOx) and the likelihood of newborns developing CH (OR 1.23 [95% CI 1.08 to 1.41]). However, there was no association between early and mid-pregnancy exposure to NOx and NO_2_ and the risk of CH (24). In a cohort study conducted at the University Clinical Centre in Tuzla focusing on autoimmune thyroid disease (AITD), five major air pollutants (PM_2.5_, NO_2_, SO_2_, CO, and O_3_) were analyzed. However, the findings indicated that the average concentrations of these pollutants were not statistically associated with an increased risk of AITD in the population of the region (25).

### Cross-sectional study

More than half of these cross-sectional studies were based on national databases from China, and seven studies utilized monitoring stations to assess pollutant exposure levels. Thyroid hormone levels were the primary outcomes in five of these studies. A study from Korea found a positive association between PM_10_ exposure and TSH levels in adults, while annual mean exposure to NO_2_ and CO was significantly associated with elevated TSH levels and reduced FT_4_ concentrations (26). In Chinese adults, increased PM_2.5_ levels were significantly negatively correlated with FT_4_ and the FT_4_/FT_3_ ratio, but positively correlated with FT_3_ levels (2). Higher exposure to PM_2.5_-bound metals was associated with lower FT_4_ and higher FT_3_ levels in pregnant women (27). Additionally, studies in pregnant women in Greece and adults in Spain found that PM_2.5_ exposure was linked to increased TSH levels, but no associations were observed between NO_2_ exposure and thyroid hormone levels (28, 29).

Three cross-sectional studies on air pollution and thyroid diseases have been conducted in China. Two of these studies utilized environmental air pollution data from the Chinese Air Quality Online Monitoring and Analysis Platform (https://www.aqistudy.cn/) (30, 31). They examined the relationship between maternal exposure to air pollutants and the likelihood of CH in offspring. Among these pollutants, O_3_ (OR 1.06 [95% CI 1.01 to 1.10]), NO_2_ (OR 1.10 [95% CI 1.02 to 1.18]), and PM_2.5_ (OR 1.02 [95% CI 1.00 to 1.03]) were significantly positively associated with the risk of CH in offspring (30, 31). However, there were no significant associations of exposure to SO_2_, CO, or PM_10_ with the risk of CH. Another study, which included a cohort of 4.9 million Chinese adults, examined the associations between exposure to PM_2.5_, PM_10_, NO_2_, SO_2_, CO, and O_3_ and the risk of TNs. The findings revealed significant linear associations between each of the six air pollutants and the risk of TNs (32).

### Case−control studies

There were four case−control studies, one of which was nested within a cohort (33–36). Out of the 13 included articles, three investigated thyroid cancer using case−control study designs. A nested case−control study in South Korea concerning thyroid cancer revealed a positive association between thyroid cancer incidence and NO_2_ exposure (OR 1.33 [95% CI 1.24 to 1.43]) and an inverse association between thyroid cancer incidence and PM_10_ exposure (OR 0.64 [95% CI 0.60 to 0.69]). These associations remained consistent in subgroup analyses (33). Two additional case-control studies on papillary thyroid cancer (PTC) conducted at the Johns Hopkins Medical Institution in the United States provided further evidence. They demonstrated a significant association between long-term exposure to PM_2.5_ and an increased diagnosis rate of PTC (34). Prolonged exposure to PM_2.5_ over 2 years (OR 1.18 [95% CI 1.00 to 1.40]) and 3 years (OR 1.23 [95% CI 1.05 to 1.44]) was significantly correlated with an increased incidence of PTC (36). Two studies included 1,990 PTC patients as the experimental group. The main difference between the two was that Crepeau et al. had a larger sample size in their control group, but their final conclusions were consistent with the other study. Furthermore, Crepeau et al. found that this association was most significant in populations with a higher median household income (34). Another case−control study in China investigated the correlation between preconception and early pregnancy exposure to environmental particulate matter and hypothyroidism during pregnancy. The study revealed that exposure to PM_2.5_ and PM_10_ during various intervals before the last menstrual period month (LMPM), including LMPM-60 days, LMPM-30 days, and all other distances before LMPM, was associated with an increased risk of hypothyroidism. Notably, the most significant associations with hypothyroidism risk were observed for PM_2.5_ (OR 1.14 [95% CI 1.10 to 1.18]) and PM_10_ (OR 1.10 [95% CI 1.07 to 1.13]) within a 250-metre buffer zone during the LMPM period (35).

### Mendelian randomization study

In Europe, a causal relationship between PM_2.5_ exposure and hypothyroidism was investigated through a two-sample Mendelian randomization study (37). That study revealed an association between exposure to increased PM_2.5_ concentrations and an increased risk of developing hypothyroidism.

## Discussion

The objective of this scoping review is to explore the relationship between exposure to air pollutants and thyroid diseases, as well as thyroid hormones, from an epidemiological perspective. A review of published studies indicates that different types of air pollution may have varying health effects. Exposure to particulate matter, particularly PM_2.5_, has been shown to impair thyroid function in pregnant women and negatively affect their offspring. However, the findings of Shang et al. suggest no association between PM_10_ exposure and the risk of CH in offspring (38). This discrepancy may be attributed to differences in study design and exposure assessment methods.

Among the 25 studies included, cohort studies were the most common, with 12 studies, followed by cross-sectional and case-control studies. In situations where randomized trials are not feasible, cohort studies are often considered one of the most reliable forms of observational epidemiological research. Cohort studies are particularly effective in establishing causal relationships by tracking health outcomes after exposure to factors such as air pollution, helping to understand how these exposures affect thyroid function or disease progression. However, they may be prone to selection bias. For example, individuals in susceptible populations may already have the disease at the start of the study, which can skew the associations being investigated. Cross-sectional studies can quickly assess the correlation between air pollution and thyroid function, but they are limited in their ability to infer causality. The limitations of case-control studies primarily arise from the selection of control groups. If there are geographical differences between the control and case groups, the study may fail to draw clear conclusions. In cases where associations exist, bias could lead to inaccurate results. Nevertheless, no single study design is flawless. A comprehensive understanding of the multifaceted relationship between air pollutants and thyroid diseases can be best achieved by integrating studies that offer complementary strengths and limitations. In the future, large-scale cohort studies with extensive exposure levels and long-term follow-up may provide the most powerful means to elucidate these associations.

The genetic background of the study subjects, as well as the potential interactions between air pollutants and genetic factors, may increase susceptibility to diseases. Therefore, these factors should be considered as confounders in research design. Thyroid hormones play a crucial role in the development of fetuses and neonates (39). In early pregnancy, since the fetal thyroid is not yet functional, the fetus relies on maternal thyroid hormones to maintain normal growth and development (40, 41). Consequently, maternal thyroid dysfunction is recognized as a known risk factor for restricted fetal development (42). This explains why researchers often focus on thyroid diseases and related hormone levels in pregnant women and neonates (41, 43, 44).

Differences exist in the methods used to assess exposure to environmental air pollutants in various studies. Qi et al.’s research, for instance, directly employed data disseminated by air quality monitoring stations for analysis (31). However, the majority of monitors involved in direct measurements are situated in urban or polluted areas (such as power plants), and not all study subjects reside in areas where monitoring stations are present. Furthermore, the data scope can be constrained by the specific years for which information is available, rendering the obtained data suitable only for coarse approximations. The land use regression (LUR) model utilized by Zhao et al. can be employed to forecast outdoor pollutant levels at the residential addresses of each participant. Building upon data derived from satellite systems at monitoring stations and further accounting for meteorological and spatial factors, the model incorporates adjustments based on geographic information such as population density, sea level, and meteorological data (45). Consequently, this approach enables the assessment of exposure levels in areas lacking air pollution monitoring, thus significantly enhancing the accuracy of exposure evaluations. This model has been validated in several studies (38, 46). Furthermore, research has estimated the average air pollutant exposure concentrations for study participants within circular buffer zones with diameters of 250 meters, 500 meters, and 750 meters based on the location of each residence, allowing for a more comprehensive analysis of the relationships (35). Exposure duration represents a common concern, necessitating assessment based on disease susceptibility and the target population. In the context of research related to thyroid cancer, the temporal scope of exposure is notably extended compared to other thyroid conditions. In the case of specific demographic groups, such as pregnant women, some researchers have structured exposure periods to encompass distinct gestational stages (22, 23).

Among the 25 studies we included, PM_2.5_ was mentioned 21 times, while PM_10_ was noted 10 times. This highlights the widespread concern about particulate matter pollution. In particular, China, where the prevalence of thyroid diseases is high and pollution is becoming increasingly severe, may prompt local researchers to investigate the potential link between air pollution exposure and hypothyroidism (47). Additionally, nitrogen oxides, such as nitrogen dioxide (NO_2_), were the second most frequently discussed pollutants, cited 13 times, followed by SO_2_, CO, and O_3_. A substantial body of epidemiological and toxicological research has shown that PM_2.5_ can enter the respiratory system through the lungs, triggering a series of pathophysiological responses, including systemic inflammation, oxidative stress, and vascular dysfunction, which can severely impact multiple bodily systems (11). Moreover, PM_2.5_ has been shown to impair thyroid function by disrupting thyroid hormone levels, potentially leading to various thyroid diseases (48–51). Animal experiments have demonstrated that PM_2.5_ can disrupt thyroid homeostasis by affecting the synthesis of thyroid hormones, but further basic research is needed to explore these mechanisms in greater detail (16).

In summary, large-scale, long-term cohort studies are needed to better understand the prolonged effects of air pollution on thyroid hormone levels and related diseases. Interdisciplinary collaboration is essential to further elucidate the complex interactions between air pollution and the endocrine system. For vulnerable populations, such as pregnant women and newborns, enhanced health protection measures should be prioritized. In high-risk areas, stricter air quality management and control of major pollutant emissions are necessary to reduce the burden of thyroid diseases. Future research should focus on elucidating how pollutants affect thyroid function through endocrine-disrupting mechanisms and quantify these effects across various exposure scenarios. A deeper exploration of these mechanisms could lead to more effective clinical and public health interventions.

## References

[1] WHO Guidelines Approved by the Guidelines Review Committee. WHO global air quality guidelines: Particulate matter (PM(25) and PM(10)), ozone, nitrogen dioxide, sulfur dioxide and carbon monoxide. Geneva: World Health Organization (2021). © World Health Organization 2021. 34662007

[2] Zeng Y He H Wang X Zhang M An Z. Climate and air pollution exposure are associated with thyroid function parameters: a retrospective cross-sectional study. J Endocrinol Invest. (2021) 44:1515–23. doi: 10.1007/s40618-020-01461-9 33159683

[3] Cohen AJ Brauer M Burnett R Anderson HR Frostad J Estep K. Estimates and 25-year trends of the global burden of disease attributable to ambient air pollution: an analysis of data from the Global Burden of Diseases Study 2015. Lancet. (2017) 389:1907–18. doi: 10.1016/S0140-6736(17)30505-6 PMC5439030 28408086

[4] GBD 2021 Risk Factors Collaborators. Global burden and strength of evidence for 88 risk factors in 204 countries and 811 subnational locations, 1990-2021: a systematic analysis for the Global Burden of Disease Study 2021. Lancet. (2024) 403:2162–203. 10.1016/S0140-6736(24)00933-4 PMC11120204 38762324

[5] Yang J Zhou M Li M Yin P Hu J Zhang C. Fine particulate matter constituents and cause-specific mortality in China: A nationwide modelling study. Environ Int. (2020) 143:105927. doi: 10.1016/j.envint.2020.105927 32619910

[6] Pope CA 3rd Coleman N Pond ZA Burnett RT. Fine particulate air pollution and human mortality: 25+ years of cohort studies. Environ Res. (2020) 183:108924. doi: 10.1016/j.envres.2019.108924 31831155

[7] Garmendia Madariaga A Santos Palacios S Guillén-Grima F Galofré JC. The incidence and prevalence of thyroid dysfunction in Europe: a meta-analysis. J Clin Endocrinol Metab. (2014) 99:923–31. doi: 10.1210/jc.2013-2409 24423323

[8] Van Uytfanghe K Ehrenkranz J Halsall D Hoff K Loh TP Spencer CA. Thyroid stimulating hormone and thyroid hormones (Triiodothyronine and thyroxine): an American thyroid association-commissioned review of current clinical and laboratory status. Thyroid: Off J Am Thyroid Assoc. (2023) 33:1013–28. doi: 10.1089/thy.2023.0169 PMC10517335 37655789

[9] Mendoza A Hollenberg AN. New insights into thyroid hormone action. Pharmacol Ther. (2017) 173:135–45. doi: 10.1016/j.pharmthera.2017.02.012 PMC5407910 28174093

[10] Ittermann T Khattak RM Nauck M Cordova CM Völzke H. Shift of the TSH reference range with improved iodine supply in Northeast Germany. Eur J endocrinology. (2015) 172:261–7. doi: 10.1530/EJE-14-0898 25452467

[11] Li Y Xu L Shan Z Teng W Han C. Association between air pollution and type 2 diabetes: an updated review of the literature. Ther Adv Endocrinol Metab. (2019) 10:2042018819897046. doi: 10.1177/2042018819897046 31903180 PMC6931138

[12] Enewold L Harlan LC Stevens JL Sharon E. Thyroid cancer presentation and treatment in the United States. Ann Surg Oncol. (2015) 22:1789–97. doi: 10.1245/s10434-014-4209-1 PMC4417105 25361888

[13] Jung KW Won YJ Kong HJ Oh CM Cho H Lee DH. Cancer statistics in Korea: incidence, mortality, survival, and prevalence in 2012. Cancer Res Treat. (2015) 47:127–41. doi: 10.4143/crt.2015.060 PMC4398120 25761484

[14] Hollowell JG Staehling NW Flanders WD Hannon WH Gunter EW Spencer CA. T(4), and thyroid antibodies in the United States population (1988 to 1994): National Health and Nutrition Examination Survey (NHANES III). J Clin Endocrinol Metab. (2002) 87:489–99. doi: 10.1210/jcem.87.2.8182 11836274

[15] Della Guardia L Shin AC. The role of adipose tissue dysfunction in PM(2.5)-induced vascular pathology. Am J Physiol Heart Circ Physiol. (2022) 322:H971–h2. doi: 10.1152/ajpheart.00156.2022 35481793

[16] Tang S Li D Ding H Jiang M Zhao Y Yu D. GLIS3 mediated by the Rap1/PI3K/AKT signal pathway facilitates real-ambient PM(2.5) exposure disturbed thyroid hormone homeostasis regulation. Ecotoxicology Environ Saf. (2022) 232:113248. doi: 10.1016/j.ecoenv.2022.113248 35093813

[17] Tricco AC Lillie E Zarin W O'Brien KK Colquhoun H Levac D. PRISMA extension for scoping reviews (PRISMA-scR): checklist and explanation. Ann Intern Med. (2018) 169:467–73. doi: 10.7326/M18-0850 30178033

[18] Janssen BG Saenen ND Roels HA Madhloum N Gyselaers W Lefebvre W. Fetal thyroid function, birth weight, and in utero exposure to fine particle air pollution: A birth cohort study. Environ Health Perspect. (2017) 125:699–705. doi: 10.1289/EHP508 27623605 PMC5382000

[19] Wang X Liu C Zhang M Han Y Aase H Villanger GD. Evaluation of maternal exposure to PM(2.5) and its components on maternal and neonatal thyroid function and birth weight: A cohort study. Thyroid. (2019) 29:1147–57. doi: 10.1089/thy.2018.0780 31298631

[20] Zhang X Huels A Makuch R Zhou A Zheng T Xia W. Association of exposure to ambient particulate matter with maternal thyroid function in early pregnancy. Environ Res. (2022) 214:113942. doi: 10.1016/j.envres.2022.113942 35870505

[21] He Q Wu M Shi Q Tan H Wei B Tang N. Association of Ozone Exposures with the risk of thyroid nodules in Hunan Province: a population-based cohort study. Environ Health. (2022) 21:65. doi: 10.1186/s12940-022-00874-8 35799180 PMC9264600

[22] Ghassabian A Pierotti L Basterrechea M Chatzi L Estarlich M Fernández-Somoano A. Association of exposure to ambient air pollution with thyroid function during pregnancy. JAMA Netw Open. (2019) 2:e1912902. doi: 10.1001/jamanetworkopen.2019.12902 31617922 PMC6806433

[23] Zhao Y Cao Z Li H Su X Yang Y Liu C. Air pollution exposure in association with maternal thyroid function during early pregnancy. J Hazard Mater. (2019) 367:188–93. doi: 10.1016/j.jhazmat.2018.12.078 30594719

[24] Harari-Kremer R Calderon-Margalit R Korevaar TIM Nevo D Broday D Kloog I. Associations between prenatal exposure to air pollution and congenital hypothyroidism. Am J Epidemiol. (2021) 190:2630–8. doi: 10.1093/aje/kwab187 34180983

[25] Izic B Husejnovic MS Caluk S Fejzic H Kundalic BS Custovic A. Urban air pollution associated with the incidence of autoimmune thyroid diseases. Med Arch. (2022) 76:115–21. doi: 10.5455/medarh.2022.76.115-121 PMC9233456 35774048

[26] Kim HJ Kwon H Yun JM Cho B Park JH. Association between exposure to ambient air pollution and thyroid function in Korean adults. J Clin Endocrinol Metab. (2020) 105: e2912–20. doi: 10.1210/clinem/dgaa338 32491176

[27] Qiu L Shen W Ye C Wu J Zheng S Lou B. Association of exposure to PM(2.5)-bound metals with maternal thyroid function in early pregnancy. Sci Total Environ. (2022) 810:151167. doi: 10.1016/j.scitotenv.2021.151167 34699824

[28] Ilias I Kakoulidis I Togias S Stergiotis S Michou A Lekkou A. Atmospheric pollution and thyroid function of pregnant women in Athens, Greece: A pilot study. Med Sci (Basel Switzerland). (2020) 8(2):19. doi: 10.3390/medsci8020019 PMC7353503 32260367

[29] Valdés S Doulatram-Gamgaram V Maldonado-Araque C Lago-Sampedro A García-Escobar E García-Serrano S. Ambient air pollution and thyroid function in Spanish adults. A nationwide population-based study (Di@bet.es study). Environ Health. (2022) 21:76. doi: 10.1186/s12940-022-00889-1 35978396 PMC9387071

[30] Shang L Huang L Yang W Qi C Yang L Xin J. Maternal exposure to PM(2.5) may increase the risk of congenital hypothyroidism in the offspring: a national database based study in China. BMC Public Health. (2019) 19:1412. doi: 10.1186/s12889-019-7790-1 31739791 PMC6862828

[31] Qi C Shang L Yang W Huang L Yang L Xin J. Maternal exposure to O(3) and NO(2) may increase the risk of newborn congenital hypothyroidism: a national data-based analysis in China. Environ Sci pollut Res Int. (2021) 28:34621–9. doi: 10.1007/s11356-021-13083-6 PMC8275538 33655476

[32] Zhang Y Wang K Qin W Jin C Song Y Jia P. Six air pollutants associated with increased risk of thyroid nodules: A study of 4.9 Million Chinese Adults. Front Endocrinol (Lausanne). (2021) 12:753607. doi: 10.3389/fendo.2021.753607 34966357 PMC8710776

[33] Park SJ Min C Yoo DM Choi HG. National cohort and meteorological data based nested case-control study on the association between air pollution exposure and thyroid cancer. Sci Rep. (2021) 11:21562. doi: 10.1038/s41598-021-00882-7 34732774 PMC8566463

[34] Crepeau P Zhang Z Udyavar R Morris-Wiseman L Biswal S Ramanathan M Jr.. Socioeconomic disparity in the association between fine particulate matter exposure and papillary thyroid cancer. Environ Health. (2023) 22:20. doi: 10.1186/s12940-023-00972-1 36823621 PMC9948306

[35] Sun Q Chen Y Ye F Liu J Liu D Ao B. Association of hypothyroidism during pregnancy with preconception and early pregnancy exposure to ambient particulate matter. Environ Sci pollut Res Int. (2023) 30:88084–94. doi: 10.1007/s11356-023-28683-7 PMC10406672 37434057

[36] Karzai S Zhang Z Sutton W Prescott J Segev DL McAdams-DeMarco M. Ambient particulate matter air pollution is associated with increased risk of papillary thyroid cancer. Surgery. (2022) 171:212–9. doi: 10.1016/j.surg.2021.05.002 PMC8688174 34210530

[37] Zhang Y Liu S Wang Y Wang Y. Causal relationship between particulate matter 2.5 and hypothyroidism: A two-sample Mendelian randomization study. Front Public Health. (2022) 10:1000103. doi: 10.3389/fpubh.2022.1000103 36504957 PMC9732245

[38] Zhang Z Kang J Hong YS Chang Y Ryu S Park J. Long-term particulate matter exposure and incidence of arrhythmias: A cohort study. J Am Heart Assoc. (2020) 9:e016885. doi: 10.1161/JAHA.120.016885 33146044 PMC7763729

[39] Sarkhail P Mehran L Askari S Tahmasebinejad Z Tohidi M Azizi F. Maternal thyroid function and autoimmunity in 3 trimesters of pregnancy and their offspring's thyroid function. Hormone Metab Res = Hormon- und Stoffwechselforschung = Hormones metabolisme. (2016) 48:20–6. doi: 10.1055/s-0035-1555878 26566101

[40] Morreale de Escobar G Obregon MJ Escobar del Rey F. Role of thyroid hormone during early brain development. Eur J Endocrinol. (2004) 151 Suppl 3:U25–37. doi: 10.1530/eje.0.151u025 15554884

[41] Salazar P Villaseca P Cisternas P Inestrosa NC. Neurodevelopmental impact of the offspring by thyroid hormone system-disrupting environmental chemicals during pregnancy. Environ Res. (2021) 200:111345. doi: 10.1016/j.envres.2021.111345 34087190

[42] Johns LE Ferguson KK Cantonwine DE Mukherjee B Meeker JD McElrath TF. Subclinical changes in maternal thyroid function parameters in pregnancy and fetal growth. J Clin Endocrinol Metab. (2018) 103:1349–58. doi: 10.1210/jc.2017-01698 PMC6018657 29293986

[43] Su YF Li C Xu JJ Zhou FY Li T Liu C. Associations between short-term and long-term exposure to particulate matter and preterm birth. Chemosphere. (2023) 313:137431. doi: 10.1016/j.chemosphere.2022.137431 36455656

[44] Martens DS Cox B Janssen BG Clemente DBP Gasparrini A Vanpoucke C. Prenatal air pollution and newborns' Predisposition to accelerated biological aging. JAMA Pediatr. (2017) 171:1160–7. doi: 10.1001/jamapediatrics.2017.3024 PMC6233867 29049509

[45] Liu C Henderson BH Wang D Yang X Peng ZR. A land use regression application into assessing spatial variation of intra-urban fine particulate matter (PM2.5) and nitrogen dioxide (NO2) concentrations in City of Shanghai, China. Sci Total Environ. (2016) 565:607–15. doi: 10.1016/j.scitotenv.2016.03.189 27203521

[46] Zhang Z Wang J Hart JE Laden F Zhao C Li T. National scale spatiotemporal land-use regression model for PM2.5, PM10 and NO2 concentration in China. Atmospheric Environ. (2018) 192:48–54. doi: 10.1016/j.atmosenv.2018.08.046

[47] Li Y Teng D Ba J Chen B Du J He L. Efficacy and safety of long-term universal salt iodization on thyroid disorders: epidemiological evidence from 31 provinces of mainland China. Thyroid. (2020) 30:568–79. doi: 10.1089/thy.2019.0067 32075540

[48] Waugh DT. Fluoride exposure and indicators of thyroid functioning: study design and data analysis considerations. J Epidemiol Community Health. (2017) 71:1226. doi: 10.1136/jech-2017-209956 28993470

[49] Kim H Kim J Kim S Kang SH Kim HJ Kim H. Cardiovascular effects of long-term exposure to air pollution: A population-based study with 900 845 person-years of follow-up. J Am Heart Assoc. (2017) 6(11):e007170. doi: 10.1161/JAHA.117.007170 PMC5721790 29118034

[50] Li R Kou X Geng H Xie J Tian J Cai Z. Mitochondrial damage: an important mechanism of ambient PM2.5 exposure-induced acute heart injury in rats. J Hazard Mater. (2015) 287:392–401. doi: 10.1016/j.jhazmat.2015.02.006 25677476

[51] Nie X Chen Y Chen Y Chen C Han B Li Q. Lead and cadmium exposure, higher thyroid antibodies and thyroid dysfunction in Chinese women. Environ pollut. (2017) 230:320–8. doi: 10.1016/j.envpol.2017.06.052 28667913

[52] Howe CG Eckel SP Habre R Girguis MS Gao L Lurmann FW. Association of prenatal exposure to ambient and traffic-related air pollution with newborn thyroid function: findings from the children's health study. JAMA Netw Open. (2018) 1:e182172. doi: 10.1001/jamanetworkopen.2018.2172 30646156 PMC6324507

[53] Li J Liao J Hu C Bao S Mahai G Cao Z. Preconceptional and the first trimester exposure to PM(2.5) and offspring neurodevelopment at 24 months of age: Examining mediation by maternal thyroid hormones in a birth cohort study. Environ pollut. (2021) 284:117133. doi: 10.1016/j.envpol.2021.117133 33894536

[54] Irizar A Txintxurreta A Molinuevo A Jimeno-Romero A Anabitarte A Álvarez JI. Association between prenatal exposure to air pollutants and newborn thyroxine (T4) levels. Environ Res. (2021) 197:111132. doi: 10.1016/j.envres.2021.111132 33839121

[55] Zhou Y Zhu Q Wang P Li J Luo R Zhao W. Early pregnancy PM(2.5) exposure and its inorganic constituents affect fetal growth by interrupting maternal thyroid function. Environ pollut. (2022) 307:119481. doi: 10.1016/j.envpol.2022.119481 35597481

